# ClusterChirp: A GPU-accelerated Web Server for Natural Language–Guided Interactive Visualization and Analysis of Large Omics Data

**Published:** 2026-02-09

**Authors:** Osho Rawal, Rex Lu, Edgar Gonzalez-Kozlova, Sacha Gnjatic, Zeynep H. Gümüş

**Affiliations:** 1 Department of Genetics and Genomics, Icahn School of Medicine at Mount Sinai, New York, NY 10029, USA; 2 Department of Immunology & Immunotherapy, Icahn School of Medicine at Mount Sinai, New York, NY 10029, USA; 3 Precision Immunology Institute, Icahn School of Medicine at Mount Sinai, New York, NY 10029, USA; 4 Windreich Department of Artificial Intelligence and Human Health, Icahn School of Medicine at Mount Sinai, New York, NY 10029, USA

**Keywords:** data visualization, GPU-acceleration, heatmap visualization, clustering, large language models, correlation networks, interactive exploration

## Abstract

Tabular datasets are commonly visualized as heatmaps, where data values are represented as color intensities in a matrix to reveal patterns and correlations. However, modern omics technologies increasingly generate matrices so large that existing visual exploration tools require downsampling or filtering, risking loss of biologically important patterns. Additional barriers arise from tools that require command-line expertise, or fragmented workflows for downstream biological interpretation. We present ClusterChirp, a web-based platform for real-time, interactive exploration of large-scale data matrices enabled by GPU-accelerated rendering and parallelized hierarchical clustering using multiple CPU cores. Built on deck.gl and multi-threaded clustering algorithms, ClusterChirp supports on-the-fly clustering, multi-metric sorting, feature search, and adjustable visualization parameters for interactive explorations. Uniquely, a natural language interface powered by a Large Language Model helps users perform complex operations and build reproducible workflows from conversational commands. Furthermore, users can select clusters to explore interactive within-cluster correlation networks in 2D or 3D, or perform functional enrichment through biological knowledge bases. Developed with iterative user feedback and adhering to FAIR4S principles, ClusterChirp empowers researchers to extract insights from high-dimensional omics data with unprecedented ease and speed. This website is freely available at clusterchirp.mssm.edu, with no login required.

## INTRODUCTION

Modern omics technologies routinely generate high-dimensional datasets, profiling tens of thousands of molecular features across hundreds or thousands of samples(1). Transcriptomic, proteomic, and metabolomic experiments capture molecular landscapes under diverse biological conditions, generating rich repositories for systems-level discovery(2). However, the rapid expansion of data generation has outpaced the capabilities of current computational infrastructure and visualization tools (3) (4) (5), creating a growing gap between data generation and biological interpretation.

Among the most pressing bottlenecks is visualization. Clustering coupled with heatmap visualizations is widely used to identify co-regulated molecular features and global patterns (6), but these methods scale poorly. Hierarchical clustering typically exhibits quadratic to cubic time complexity (7), requiring on the order of nearly 50 million pairwise distance calculations for just 10,000 genes, and rendering real-time, interactive visualization infeasible on standard hardware. Web-based tools add further constraints, including session timeouts, server-side processing limits, and memory caps, often forcing users to downsample data and risk missing rare but biologically meaningful patterns. Accessibility compounds the problem, as many high-performance tools require command-line expertise or specialized syntax, limiting adoption by researchers without extensive computational training.

Even when visual patterns are detected, downstream interpretation often involves fragmented workflows that require exporting gene lists, converting identifiers, and manually querying external enrichment tools. These steps slow analysis, increase the potential for error, and lead to incomplete biological insights. Numerous heatmap visualization tools have been developed, including web-based platforms such as (8) (9) (10) (11) (12) (13), which offer varying degrees of interactivity and enrichment integration. While impactful and widely used, these tools often face limitations in scalability, session stability, computational throughput, or long-term availability when applied to large-scale omics datasets. Desktop applications and programming libraries, including MATLAB, R and Python packages such as ComplexHeatmap (14), pheatmap (15), seaborn (16), and plotly (17)provide greater flexibility, but remain constrained by memory limits, licensing restrictions, or usability barriers. As a result, large-scale omics studies are frequently limited to static visualizations, slow exploratory cycles, or partial functional interpretation.

These limitations impede biological discovery. Downsampling can mask important functional subgroups, while static or slow visualizations hinder dynamic hypothesis generation, such as interactively adjusting clustering parameters, filtering by metadata, or updating visualizations in real time. Functional interpretation and within-cluster relationship analyses, including correlation networks, often require separate tools, further fragmenting the workflows and increasing cognitive overhead. Together, these challenges highlight an unmet need for tools that support real-time, large-scale visualizations integrated with biological context that are accessible by researchers of all computational skill levels.

To reduce the usability barriers, large language models (LLMs) offer a promising complement to computational improvements. As visualization systems grow in complexity, natural language interfaces powered by LLMs can simplify interaction, by allowing users to issue high-level commands, such as “*cluster genes using Pearson correlation*”, instead of navigating complex menus or scripting interfaces (18) (19). While LLMs do not not address scalability directly, they improve accessibility for non-programmers and accelerate exploratory workflows for experienced users (20) (21)

To address both computational and usability challenges, we developed **ClusterChirp**, a GPU-accelerated web-based platform for real-time interactive exploration of omics datasets containing thousands of samples and tens of thousands of features. ClusterChirp is designed to scale to datasets of close to 10 million cells while maintaining smooth interactive performance, and introduces several innovations: i) GPU-based rendering through WebGL (using deck.gl for heatmaps and Sigma.js/Three.js for networks) that maintains 60fps interaction with million-point displays, the GPU handles visualization only, while computation runs on the server; ii) a parallelized hierarchical clustering engine that distributes distance matrix calculations across CPU cores, reducing clustering runtimes from hours to minutes; iii) an AI-powered natural language interface to support conversational analysis; iv) within-cluster pairwise correlation visualizations to reveal internal patterns and subclusters as interactive network graphs in 2D or 3D layouts; and v) seamless integration with biological knowledgebases to support real-time functional interpretation. By bridging the gap between high-throughput data generation and biological interpretation, ClusterChirp enables researchers to fully leverage the scale and complexity of modern omics datasets, while broadening accessibility across computational skill levels. ClusterChirp is available as a freely accessible public web server.

## MATERIALS AND METHODS

### User-Centered Design and Prototyping.

We developed ClusterChirp using an iterative, user-centered design approach involving 10 domain practitioners in visual omics exploration within immunology, genomics and bioinformatics. Initial interviews with 5 experts revealed key pain points, computational bottlenecks, and usability issues limiting large-scale interactive analyses. Based on this feedback, we i) adopted basic design elements from an established heatmap visualization tool, Clustergrammer (13), and ii) derived core functional and usability requirements. To prioritize features within core visualizations, interactions and workflows, we utilized low-fidelity prototypes, followed by high-fidelity interactive prototypes in React (22) and TypeScript (23). In biweekly semi-structured evaluation sessions, the domain experts performed representative analysis tasks, providing real-time verbal feedback that guided user interface (UI) refinements, focusing on accessibility and functionality. These included a natural language interface powered by large language models (LLMs), and correlation network-based subcluster exploration. The final prototype was evaluated by 5 additional researchers from Mount Sinai and Brown University to ensure the platform directly addresses the needs for large and complex datasets.

### FAIR4S Principles and Software Availability.

To ensure long-term sustainability, we adhered to Findable, Accessible, Interoperable, Reusable for Research Software (FAIR4S) principles during development. Findable: ClusterChirp is available at a stable URL, with version source code hosted on GitHub (frontend: https://github.com/GumusLab/HeatmapFrontend; backend: https://github.com/GumusLab/HeatmapBackend) Accessible: The platform is freely accessible via modern web browsers without registration or installation, and the source code is distributed under the APGL-3.0 license. Interoperable: ClusterChirp supports standard tab-delimited text formats commonly used in transcriptomic and other omic workflows, exports clustered data matrices (TSV) and publication-ready figures (PDF). Reusable: Detailed documentation, tutorials, and example datasets with expected outputs are provided to support transparent reuse and reproducibility.

### Interactive Heatmap Visualization.

To support real-time visualization of large heatmaps with interactive dendrograms and integrated metadata, we implemented GPU-accelerated rendering using the WebGL-based framework deck.gl (24). We built the heatmap as a custom React component, with matrix cells rendered using ScatterLayer, and row and column layers rendered using TextLayer. To optimize performance, matrix values are stored as Float32 arrays for efficient GPU data transfer, and viewport-based culling renders only visible regions during visual navigation. User interactions including tooltips, pan, zoom, and dynamic search highlighting are managed through deck.gl event handlers, while computationally intensive tasks such as sorting and filtering are offloaded to Web Workers to maintain UI responsiveness. The GPU is used strictly for rendering; all clustering and correlation calculations run on server CPUs (see Backend Architecture and Clustering Implementation).

### Natural Language Interface (AI Chatbot).

To lower barriers for non-technical users and accelerate expert workflows, ClusterChirp includes a natural language chatbot powered by GPT-4o-mini (version gpt-4o-mini-2024–07-18) (https://platform.openai.com/docs/models/gpt-4o-mini). The chatbot interprets commands, captures current heatmap visualization state (filter, sort, cluster settings) and translates the commands to frontend or backend actions. Commands are classified as: i) view-only front-end operations (e.g. adjusting opacity or sorting); ii) backend filtering and/or clustering (e.g. filtering by metadata fields); and iii) enrichment queries, which invoke curated gene sets before applying filters to the dataset. Frontend commands update visualization layers directly, while backend commands are processed via a Django API endpoint. For each command, a dynamic prompt is constructed, including the current query, session command history, active clustering state, filtering settings, and session-specific metadata (session_id_metadata.json). This enables context-aware processing, allowing sequential commands to build on prior operations. The LLM returns a standardized JSON action object, which is validated against dataset parameters before execution. Invalid values trigger error messages listing available alternatives, and a rule-based fallback parser ensures functionality when the LLM API is unavailable.

### Correlation Network Visualization.

For a user-selected cluster, ClusterChirp supports analyzing co-expression patterns within it. Towards this end, it calculates pairwise similarity between cluster members and then plots correlation networks. Users have the option to plot these networks in 2D or 3D. To generate these within-cluster networks, the tool first removes features with >20% missing values (25), and only retains the high-variance ones among the remaining features (default: top 75%) (26). Pairwise similarity is calculated using Pearson correlations. To scale these computations, feature pairs are processed in parallel using joblib (https://joblib.readthedocs.io/) and Numba JIT compilation (https://numba.pydata.org/). Statistical significance is assessed via Fisher’s z-transformation (n nodes > 30) or t-distribution approximation. Default thresholds filter correlations with |r| ≥ 0.7 (27)and p < 0.05 (both user-customizable); top N correlations (default 100,000) are retained in float32 format and returned as a JSON file including correlation coefficients, p-values, and sample size. For network layouts in 2D, the tool uses Sigma.js 3.0 (https://www.sigmajs.org/) with WebGL rendering and the ForceAtlas2 (28) force-directed layout algorithm to group nodes by correlation strength, with node coloring based on connectivity degree. For network layouts in 3D, ClusterChirp uses Three.js (https://threejs.org/) with WebGL rendering, with Leiden clustering (29) to identify node communities, where each community is mapped to distinct regions of a 3D spherical surface and nodes are colored by cluster membership. Large networks (>1,000 nodes) are computed in Web Workers to prevent UI blocking. Both layouts support real-time interactivity, including dynamic node search, autocomplete and highlighting, with GPU buffer updates.

### Gene-set enrichment analysis (Enrichr integration).

Users can perform gene set enrichment analysis on any cluster by selecting it directly from the heatmap, with results generated via the Enrichr tool (30–32). When a user selects a cluster, gene identifiers are extracted and harmonized to standardized HUGO symbols. For Olink Target proteomics panels (33), protein names are mapped using a predefined dictionary (https://olink.co), The standardized gene list is then submitted to Enrichr, and results are displayed in a new browser tab.

### Tutorials.

A four-module tutorial series covers basic navigation and heatmap interaction; natural language interaction; Enrichr-based enrichment analysis, and correlation network exploration in 2D and 3D. Each module includes step-by-step instructions, annotated screenshots, demo videos, example datasets, and troubleshooting tips for common issues. The tutorials are fully responsive for use across desktops, tablets, and mobile devices.

### Data Security and Session Management.

ClusterChirp uses secure, session-based data handling implemented in Django on Mount Sinai’s HIPAA-compliant Minerva server environment (34). Uploaded datasets are assigned a unique session ID and stored temporarily, ensuring isolation between users and sessions. Metadata (e.g. categorical annotations) are extracted and stored in lightweight JSON files to enable fast queries without loading full matrices. Django’s session framework maintains a secure, persistent state, and temporary files are automatically removed on session expiration, ensuring data privacy and storage efficiency.

### Backend Architecture and Clustering Implementation.

ClusterChirp backend is built on Django 4.2 (https://www.djangoproject.com/) with Django REST Framework 3.15.2, utilizing pandas 2.0.3 (https://pandas.pydata.org/) and NumPy 1.24.4 (https://numpy.org/) for core data handling. Hierarchical clustering is performed via clustergrammer-py (13) integrated into custom REST API endpoints. To scale to large datasets (>20,000 features), pairwise distance computations are parallelized using joblib (https://joblib.readthedocs.io/) with adaptive caching (default 500 MB) and block-based computation to prevent memory overflow. This approach distributes the sequential O(n^2^) computation across available hardware resources (see Results for performance benchmarks). Users can select different distance metrics (Euclidean, Manhattan, Pearson), linkage methods (complete, average, single) via the API. Users can also select hierarchical groupings at multiple cutoff thresholds (0.0 to 1.0 in increments of 0.1) to explore dendrogram structures at different levels of granularity.

### Natural Language Interface Evaluation.

We benchmarked the LLM interface using 45 commands derived from Clusterchirp supported operations: filtering (n=20), clustering (n=14), and sorting (n=11) (Supplementary Table S1). Success required correction without manual intervention; commands that succeeded after rewording were recorded as requiring rephrasing. API fallback rate captured GPT-4o-mini failures, and response time was measured from command submission to completion of the requested operation. Testing was performed on the GU16–257 bladder cancer immunotherapy dataset (35), comprising 77 plasma proteins measured across 196 samples. The dataset includes six metadata fields: PatientID (~50 unique patients), Timepoint (C1D1, C3D1, C8D1, C12D1), Response (Yes, No, NE), Gender (M, F), Race (white, asian, black, unknown), and Ethnicity (non_hispanic, hispanic, unknown). The interface achieved an overall success rate of 95.66%, with average response times under 2 seconds (Supplementary Table S2).

## RESULTS

We present ClusterChirp, a web-based platform designed to overcome computational and usability bottlenecks in large-scale omics data exploration. The platform integrates GPU-accelerated rendering, parallelized clustering workflows, and a natural language chatbot to enable real-time analysis of high-dimensional datasets.

### User-Centered Design Process.

To establish design requirements, we conducted structured interviews with five domain experts spanning cancer immunology, genetics, and bioinformatics. These interviews identified three recurring limitations of existing tools: i) the need to downsample large datasets, risking loss of biologically important patterns; ii) processing delays exceeding 10 minutes for routine clustering tasks; and iii) limited support for metadata-aware filtering. Guided by these requirements, we engineered ClusterChirp to prioritize scalability, responsiveness, and usability, and then evaluated iterative versions of the platform with five additional experts.

### User Interface and Data Integration Workflow.

**Figure 2.**
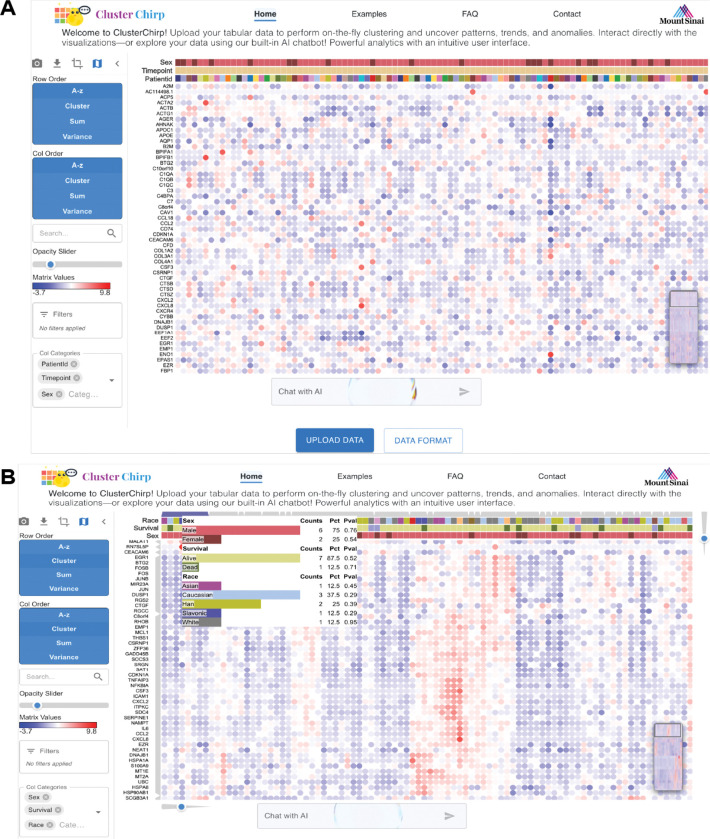
ClusterChirp web interface. **(A)** ClusterChirp homepage displaying an example dataset. The top navigation bar includes tabs for Home, Examples (to explore example datasets), FAQ (for detailed tutorials), and Contact. The left panel features controls for row and column ordering (alphabetical, cluster, sum, variance), searching specific row variable, opacity adjustment, matrix value scale, and filters. The Filters section is populated when users type commands in the AI chat box; for example, requesting “filter top 100 most variant rows” adds a row filter, and column filters appear similarly when specified. Dropdown menus for column and row categories allow users to select up to three annotation categories to display as metadata bars on the heatmap; these dropdowns are automatically populated by detecting categories in the uploaded data. Here, PatientId, Timepoint, and Sex are selected as column categories and displayed as color bars above the heatmap. The “Chat with AI” interface at the bottom enables natural language queries. Users can upload data via the “Upload Data” button or view the required input format via the “Data Format” button. **(B)** Heatmap after hierarchical clustering is applied. Cluster layers appear on top for columns and on the left for rows, with corresponding dendrogram sliders at the bottom (rows) and top right (columns). Users can adjust the slider to traverse 11 hierarchical depths, visualizing clusters at different resolutions from general to refined. Hovering over a specific cluster displays a metadata info box showing the composition for each selected annotation category. Here, the info box shows counts, percentages, and p-values for Sex (6 Male, 2 Female), Survival (7 Alive, 1 Dead), and Race (1 Asian, 3 Caucasian, 2 Han, 1 Slavonic, 1 White) within the selected cluster.

The ClusterChirp Home Page (Figure 2A) consists of four integrated components: a top navigation bar providing access to tutorials and pre-loaded example datasets; a left control panel for interactive data manipulation; a central workspace displaying an interactive heatmap that updates in real-time; and a bottom panel hosting an integrated AI assistant. Users can upload data through an “Upload Data” button, or review input file structure and metadata requirements through a “Data Format” button.

### Missing Value Handling and Imputation.

Upon data upload, ClusterChirp automatically performs data validation and detects missing values. If no missing values are present, clustering proceeds immediately, and a full-screen heatmap is rendered with integrated metadata annotations and dendrograms. If missing values are detected, users are presented with summary statistics describing missingness patterns, and offered multiple imputation strategies, including mean or median replacement, k-nearest neighbors (KNN), iterative imputation, matrix factorization, correlation-based methods, and random forest approaches. An auto-select option recommends an imputation method based on dataset characteristics (e.g. missing data percentage, distribution patterns), enabling rapid preprocessing.

### Core Data Visualizations and Manipulations.

The control panel provides tools for data manipulation, including row and column sorting (alphabetical, sum, or variance); hierarchical clustering, feature (row ID) search and highlighting, opacity adjustment to emphasize extreme values, and metadata selection.

#### Real-time Hierarchical Clustering.

ClusterChirp supports hierarchical clustering of rows and/or columns using multiple distance metrics (cosine, Euclidean, Manhattan, Pearson) and linkage methods (complete, average, single). As clustering requires computation of an n×n distance matrix (O(n^2^) memory); systems with 16 GB RAM typically support datasets up to approximately 30,000 rows, with ~20,000 rows recommended as a conservative guideline for routine usage. Following clustering, the heatmap is automatically reordered to reflect the hierarchical structure, and dendrograms are displayed adjacent to the heatmap (see Figure 2B, where column dendrograms are on top, and row dendrograms are on the left). Interactive sliders enable users to adjust clustering depth for rows and columns, facilitating exploration across hierarchical levels (see Figure 2B, sliders positioned on the right of and below the heatmap). Hovering over dendrogram branches reveals feature distributions across metadata categories.

#### Metadata Integration and Display.

ClusterChirp automatically extracts metadata from uploaded datasets and displays them as colored annotation layers (see Figure 2A). Column metadata appear as horizontal colored bars above the heatmap; row metadata are displayed as vertical colored bars to the left. Up to three metadata categories each for rows and columns can be displayed simultaneously, which can be updated dynamically via dropdown menus in the control panel.

#### Interactive Sorting and Organization.

Multiple sorting options are available for both rows and columns, including alphabetical, sum-based, and variance-based ranking. Double-clicking a metadata label triggers instant reordering of the heatmap. For example, in Figure 2B, clicking on the category label “Sex” reorganizes the samples into male and female groups, revealing metadata-associated patterns in real time.

#### Search and Opacity Controls.

A search box enables rapid identification of specific row IDs using autocomplete suggestions. Upon selection, the heatmap automatically zooms to and highlights the corresponding row. An opacity slider adjusts cell transparency to enhance contrast among data values and to facilitate identification of extreme values, with adjustable values ranging from 0.5 to 3.0.

#### Export and Navigation Tools.

ClusterChirp includes buttons to capture high-resolution PDF snapshots suitable for publication; to download processed (filtered or transformed) datasets as TSV files; cropping regions of interest and patterns for focused explorations; and turning on/off a minimap view of the entire heatmap. Users can click and drag a viewpoint indicator within the minimap to instantly update the main heatmap view to the selected regions, providing spatial context and enabling rapid global navigation.

### Natural Language Chatbot and AI-Supported Interaction.

To improve accessibility, ClusterChirp includes a conversational chatbot powered by an LLM that interprets and executes complex commands in natural language, such as “cluster genes using Pearson correlation and average linkage” and “show only male samples”. The chatbot supports a wide range of operations, including dynamic filtering, clustering, sorting, feature selection, search, and visualization parameter adjustment operations, with applied filters appearing as removable tags (e.g., “Sex: Male”) in the control panel. Guided command suggestions are organized by functional categories, and command history is preserved across sessions to support iterative exploration.

The LLM chatbot supports filtering, clustering and sorting operations. Users can filter rows or columns based on metadata (e.g., “show only female samples”), combine multiple conditions (e.g., “show female responders”), or keep only high-variance rows. Clustering can be applied to rows or columns using multiple distance metrics (Pearson, Euclidean, Manhattan, Cosine) and linkage methods (Average, Complete, Single). Sorting organizes data alphabetically, by variance, or by sum. The chatbot commands range from simple queries (e.g., “show only male samples”) to multi-step operations (e.g., “cluster genes using Pearson correlation and average linkage”)(Supplementary Table S1). Benchmarking demonstrated high execution accuracy across supported operations, low fallback rates, and minimal need for command rephrasing (Supplementary Table S2; see Natural Language Interface Evaluation in Methods). By combining guided command suggestions, dynamic filter management, and context-aware processing, the chatbot offers a reliable and efficient alternative to traditional GUI interactions.

### Gene Filtering by Biological Pathways.

Using the LLM chatbot, users can filter genes based on biological pathways and functional categories. ClusterChirp integrates pathway databases (KEGG, Reactome, WikiPathways, MSigDB), supporting pathway discovery, functional filtering, and targeted pathway selection. For exploratory analysis, users can list pathways by category (e.g., “list immune pathways” or “show cancer pathways” ) to identify relevant options. Broad queries (e.g., “show immune genes” or “metabolism genes”) automatically subset and re-cluster the dataset, while targeted queries for specific pathways or identifiers (e.g., “filter by pathway KEGG_MAPK_signaling”), generate focused heatmaps that reveal pathway-specific patterns that may not be apparent in the full dataset..

### Correlation Network Visualization.

For selected clusters, ClusterChirp enables downstream within-cluster correlation network analysis. When users select a cluster from the dendrogram, a dialog with cluster statistics and analysis options pops up; selecting network generation leads to the 2D network interface where nodes represent features and edges denote statistically significant pairwise correlations among cluster members. Nodes are color-coded by their connectivity (dark blue for high connectivity nodes (>10 connections); medium blue for moderate connectivity (5–10 connections), and light blue for low connectivity (<5 conditions)). Network controls include row ID search, and label toggles. Optional Leiden clustering identifies community structure, which can be visualized in 3D to reveal tightly connected gene modules that may represent functional units or co-regulated pathways within the cluster under exploration.

### Integration with Biological Knowledge Bases.

**Figure 3.**
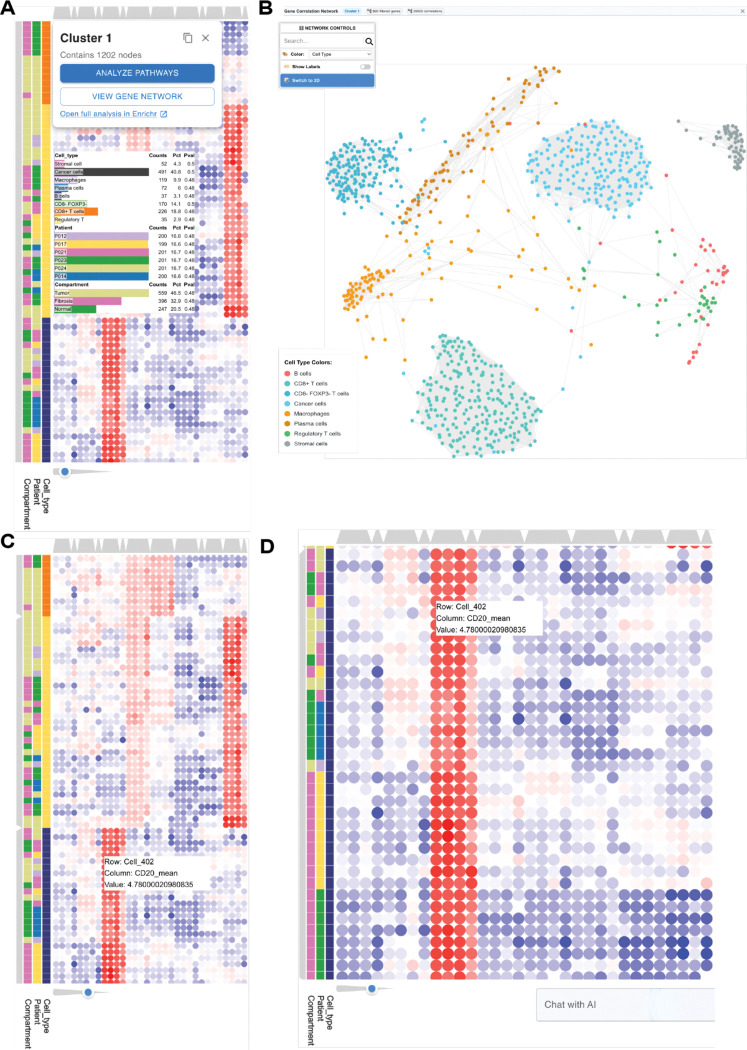
Spatial proteomics analysis reveals distinct cell type signatures in tumor microenvironment. To demonstrate ClusterChirp utility for high-dimensional spatial proteomics data exploration, we analyzed a multiplexed immunohistochemistry (mIHC) dataset comprising 1,202 single cells from six cancer patients. The dataset contained measurements of eight protein markers (CD20, CD3, CD68, CD8, FOXP3, MZB1, PanCK, αSMA) quantified at four intensity levels (p1, p5, p9, mean) per marker, producing 32 features per cell across eight annotated cell types. **(A)** Hierarchical clustering heatmap with interactive cluster selection. Hovering on a dendrogram branch opens an info box displaying cluster statistics (cell counts, proportions, p-value) stratified by Cell_Type, Patient, and Compartment annotations. Clicking on the dendrogram branch opens up a dialog box in the upper left. Users can directly launch pathway enrichment analysis via Enrichr (30–32) or visualize the cluster as a gene correlation network by clicking on the corresponding buttons in this dialog box. Red indicates high expression; blue indicates low expression. **(B)** Force-directed network visualization of all 1,202 cells, accessible via the “View Gene Network” button. Nodes represent individual cells colored by cell type, with spatial positioning determined by marker expression similarity. The network successfully separates distinct cell populations. **(C)** Dendrogram depth slider enables multi-resolution cluster exploration. The slider (bottom left, blue indicator) allows users to traverse 11 hierarchical levels, revealing progressively finer cell type distinctions at deeper cuts. With the dendrogram at level 5 it shows different cell types in different clusters. Hovering over cells displays precise values (inset: Cell_402, CD20_mean = 4.78). **(D)** Subset analysis of B cells isolated using ClusterChirp crop functionality. The cropped view shows high CD20 expression (red columns) across all cells, confirming B cell identity.

Functional enrichment analysis is supported through direct integration with the Enrichr tool (30–32). Users can submit selected gene or protein lists with a single click (see Figure 3B); protein identifiers are automatically mapped to HUGO gene symbols prior to enrichment.

### Platform Scalability, Security and Performance.

ClusterChirp is deployed on Mount Sinai’s HIPAA-compliant Minerva infrastructure (34), with datasets stored temporarily with unique session identifiers to ensure data isolation and prevent cross-contamination between users.

Benchmarking against five widely used web-based tools demonstrated that ClusterChirp supports larger datasets with greater interactivity (see Table 1). These tools include Morpheus (Broad Institute) (8), NG-CHM (MD Anderson) (9), Heatmapper2 (Wishart Lab) (10), ClustVis (11), and Clustergrammer (13). For benchmarking, we used two datasets : Dataset 1 (5,000 × 2,000; 10 million cells), a synthetic matrix designed to test scalability at documented tool limits, and Dataset 2 (10,909 proteins × 108 samples; ~1.2 million cells), a real-world mass spectrometry proteomics dataset. For Dataset 1, ClusterChirp completed clustering in ~3 minutes, Heatmapper2 in ~30 seconds, and NG-CHM in 7+ minutes. Morpheus remained incomplete after 12+ minutes, ClustVis rejected the file due to size limits, and Clustergrammer returned a 502 server error. For Dataset 2, ClusterChirp completed in 30 seconds and Heatmapper2 in ~25 seconds, while NG-CHM and ClustVis rejected the dataset due to row limits, Morpheus failed, and Clustergrammer returned server errors. Notably, while Heatmapper2 achieved fast clustering by running computations client-side via WebAssembly, avoiding network latency entirely; however, it generates static images that prevent interactive exploration. ClusterChirp performance advantage derives from its parallelized pairwise distance computation which yields over a 3-fold speedup for datasets with >20,000 features compared to sequential processing, without loss of accuracy (see Methods). This eliminates the need for researchers to downsample such large datasets, a core advantage that prevents the loss of important biological patterns. We further tested ClusterChirp performance with datasets ranging from small gene panels (100 genes × 50 samples) to large-scale omics datasets (23,563 genes × 478 samples; ~11 million cells). For visualization, the platform maintained 60fps pan/zoom interactivity up to 10 million cells, with frame rates declining beyond that due to browser memory constraints. For computation, clustering completed in minutes rather than hours across all tested dataset sizes. To ensure broad accessibility, we validated cross-browser compatibility on Chrome, Safari, and Firefox.

### CASE STUDIES.

We demonstrate ClusterChirp’s capabilities through two detailed case studies, accessible for interactive exploration on the platform’s web portal.

#### Case Study 1: Spatial Proteomics Analysis Reveals Distinct Cell Type Signatures in Tumor Microenvironment.

To demonstrate the utility of ClusterChirp in high-dimensional spatial proteomics data exploration, we used publicly available multiplexed immunohistochemistry (mIHC) data from Buckup et al(36), comprising single-cell protein measurements from six cancer patients. In this study, cells were classified into eight types (Cancer cells,CD8+ T cells, CD8− FOXP3- T cells, Regulatory T cells, B cells, Plasma cells, Macrophages, Stromal cells) based on exclusive lineage marker emxpression. To generate a representative subset, we selected ~200 cells per patient using proportional sampling with a separation score prioritizing cells most typical of their assigned type, yielding 1,202 cells with 32 features (8 markers × 4 intensity metrics) across three tissue compartments.The dataset contains measurements of eight protein markers (CD20, CD3, CD68, CD8, FOXP3, MZB1, PanCK, aSMA) quantified at four intensity levels (p1, p5, p9, mean) per marker.

First, hierarchical clustering separated all eight cell types into distinct clusters (Figure 3A), grouping related immune cell subtypes (CD8+ T cells, FOXP3+ T cells, CD8+ FOXP3+ T cells, and CD3+ T cells) together, while cancer cells (PanCK+), B cells (CD20+/MZB1+), macrophages (CD68+), and stromal cells (aSMA+) formed separate branches. The heatmap generated by ClusterChirp displayed clear differential expression patterns, with high expression values in red and low expression in blue. Clicking on cluster layer branches opens an interactive dialog displaying cluster statistics stratified by annotation categories, with options for pathway enrichment analysis via the external Enrichr tool (30–32) or visualization of within-cluster correlation network. Network visualization reveals cell type community structure, where the force-directed network view (Figure 3B) displays all 1,202 cells as nodes colored by cell type, with spatial positioning determined by marker expression similarity. This visualization clearly shows the separation of distinct cell populations into communities, with T cell subtypes clustering together while B cells, cancer cells, macrophages, plasma cells, and stromal cells forming separate groups.

Next, we used subsetting and re-clustering the selected subset options to verify that cluster assignments reflect true biological differences rather than technical artifacts. Towards this end, to identify both major cell lineages and subtle subpopulations within the tumor microenvironment, we explored the heatmap at multiple scales using the dendrogram depth slider (Figure 3C). Traversing across 11 hierarchical levels revealed progressively finer cell type distinctions at deeper cuts. Then, we explored specific populations by using the crop functionality, selecting B cells for detailed examination (Figure 3D). The cropped view showed high CD20 expression across all cells in this cluster, confirming B cell identity.

In this case study, having the options to adjust the dendrogram depth, and analyze subsets of specific cell populations through co-expression network views made it easy to visually confirm that the separations we were seeing reflected actual biological differences in protein expression rather than technical artifacts or batch effects. This interactive validation approach is especially useful for spatial proteomics data, where visual inspection of marker expression patterns is essential for confirming cell type assignments and identifying potential misclassifications.

#### Case Study 2. Natural language-assisted Analysis of Bladder Cancer Treatment Response Biomarkers.

We analyzed plasma protein data from the GU16–257 bladder cancer immunotherapy trial (35)**,** with data kindly provided by the study investigators. Data were generated using the Olink Immuno-Oncology panel (92 proteins; 77 retained after QC filtering) and provided as log2-transformed Normalized Protein eXpression (NPX) values, measured at multiple treatment cycles (C1D1, C3D1, C8D1, and C12D1) across 196 patient samples.

**Figure 4.**
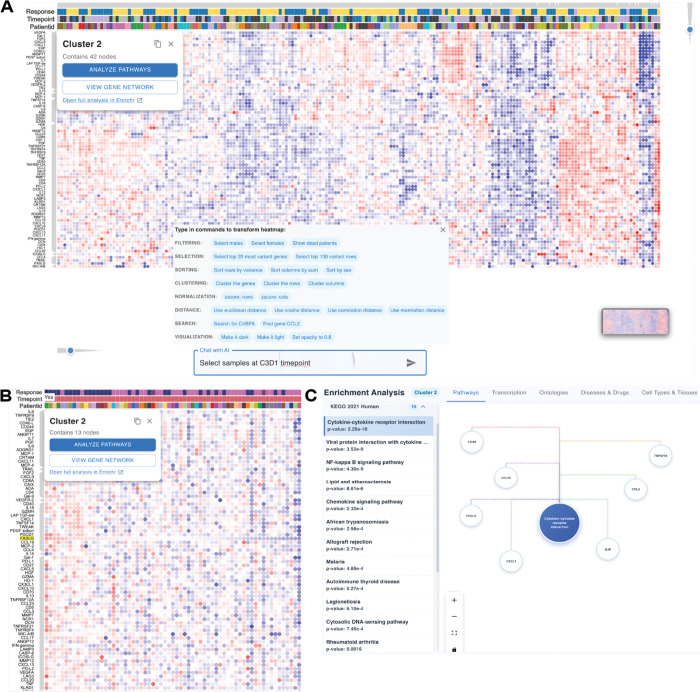
Natural language-guided analysis of treatment response biomarkers in bladder cancer plasma proteomics data. Data from the GU16–257 bladder cancer immunotherapy trial (35) comprising 77 plasma proteins measured across 196 samples at multiple treatment cycles (C1D1, C3D1, C8D1, C12D1). **(A)** Hierarchical clustering of the full dataset with cluster selection dialog (Cluster 2, 42 genes). A command guide popup displays available natural language operations organized by category: Filtering (e.g., “Select males”), Selection (e.g., “Select top 100 variant rows”), Sorting (e.g., “Sort rows by variance”), Clustering, Normalization, Distance metrics, Search, and Visualization options. The “Chat with AI” box shows an example command “Select samples at C3D1 timepoint”. Metadata bars on top display Response status, Timepoint, and PatientId. **(B)** Filtered heatmap showing only C3D1 timepoint samples after AI-assisted filtering. Samples are sorted by FASLG expression (descending), revealing enrichment of responders (dark blue in Response metadata bar) among samples with high FASLG expression. Cluster 2 now contains 13 genes in the filtered view. Clicking on a cluster opens a dialog with options to analyze pathways, view gene network, or open full analysis in Enrichr (30–32). **(C)** Pathway enrichment analysis results for Cluster 2 using KEGG 2021 Human database. The top enriched pathway is Cytokine-cytokine receptor interaction (p-value = 2.25e-10). The network visualization (right) shows cluster genes (CD40, CCL19, FASLG, CXCL1, IL1B, CCL4, TNFSF14) connected to this pathway.

Following data upload, hierarchical clustering automatically organized the 77 proteins into distinct co-expression clusters (Figure 4A). The dendrogram on the left margin showed clear separation between clusters, with Cluster 2 containing 42 genes displaying coordinated expression patterns. We customized the visualization by selecting metadata bars to display the heatmap, showing response status (responder/non-responder), timepoint, and patient ID for each sample, with red coloration indicating higher protein expression and blue indicating lower expression. To examine protein expression at a specific treatment timepoint, we used ClusterChirp natural language interface by typing “Select samples at C3D1 timepoint” into the command box. The system filtered the heatmap in real time and displayed data only from cycle 3 day 1 samples (Figure 4B), demonstrating how conversational commands can replace complex manual filtering operations. Next, double-clicking on the *FASLG* gene sorted all samples by its expression values in descending order. This revealed that patients who achieved complete clinical response (indicated by dark blue bars in the response metadata track) were enriched on the left side of the heatmap where *FASLG* expression was highest (Figure 4B), aligning with published findings from this trial linking high *FASLG* expression to better treatment outcomes.

Next, to better understand the biological functions of genes co-expressed with *FASLG*, we selected Cluster 2 (Figure 4B) and clicked on “ANALYZE PATHWAY”.This submitted the cluster genes list to the external Enrichr GSEA tool (30–32), which performs enrichment analysis against its curated library collection. Users can scroll through results from different libraries available via Enrichr to identify relevant pathways. From the KEGG 2021 Human pathway database (Figure 4C), the top enriched pathway was cytokine-cytokine receptor interaction (p-value = 2.25e-10), indicating that proteins co-expressed with *FASLG* are predominantly involved in immune cell communication and signaling. Other enriched pathways included *viral protein interaction with cytokines*, *TNF signaling*, and *chemokine signaling pathways*, collectively suggesting coordinated immune and inflammatory responses.

In this case study, the complete workflow was performed without writing any code, including hierarchical clustering and metadata visualization through natural language-guided filtering, interactive sorting, cluster selection, and pathway enrichment analysis. ClusterChirp enabled rapid hypothesis generation on treatment response biomarkers through intuitive visual exploration and on-the-fly biological interpretation.

## Discussion

ClusterChirp is a GPU-accelerated, LLM-supported web platform that leverages parallelized clustering to address major computational and usability challenges in visual exploration of large -omics datasets. It combines real-time heatmap creation, hands-on exploration of correlation networks and gene set enrichment in one seamless system, so users can work with full datasets without having to reduce their size, lose important details, or switch between different software programs. With its natural language chatbot, users at any computational skill level can easily run advanced analyses just by typing commonly used questions, such as asking to cluster genes by expression levels, instead of navigating complex operations or command-line instructions. This gives non-programmers the same strong capabilities as computational experts, but without the steep learning requirements or barriers that often keep beginners out. Additionally, its built-in pathway analysis through Enrichr (30–32), allows researchers to dive straight into biological insights from their results without copying data or opening extra apps. Rather than redirecting users to an external website, ClusterChirp calls the Enrichr API directly and displays results within the interface, keeping the workflow seamless. Overall, by blending user-friendly interfaces with top-tier computing power, ClusterChirp sets a new standard for tools that let every researcher, no matter their technical skills, perform interactive, real-time studies on massive omics datasets. To maximize usability while ensuring data security, ClusterChirp is freely available at https://clusterchirp.mssm.edu, hosted on Mount Sinai’s HIPAA-compliant Minerva infrastructure.

This tool should be considered in the context of its limitations. First, the current hierarchical clustering implementation has computational constraints that limit analysis of very large datasets such as single-cell sequencing data containing hundreds of thousands of rows. Future development will focus on integrating computationally simpler clustering algorithms to address this limitation. Second, while the natural language interface handles most common commands effectively, highly complex or ambiguous queries may be misinterpreted, requiring users to rephrase their requests. Third, pathway enrichment functionality depends on the Enrichr API (30–32); if this service is unavailable, enrichment analysis will be temporarily affected. Fourth, tool performance is dependent on Sinai’s Minerva computing infrastructure; server downtime or high load may impact response times. Finally, as with any tool that accepts user-uploaded data, incorrectly formatted uploads may cause errors, though the platform provides format guidelines to minimize this issue.

## Supplementary Material

Supplement 1

## Figures and Tables

**Figure 1.**
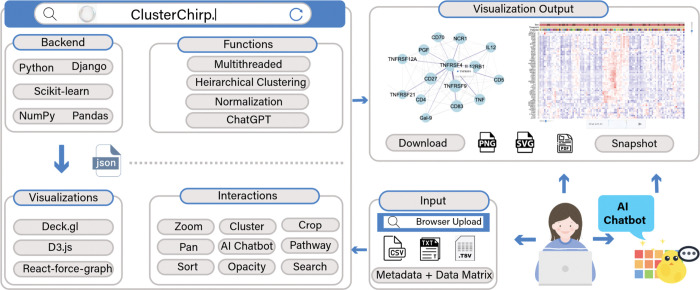
Overall architecture of ClusterChirp.

**Table 1.** Benchmarking ClusterChirp with other web-based tools.

Feature	Morpheus	NG-CHM	Heatmapper2	ClustVis	Clustergrammer	ClusterChirp
Availability	✓	✓	✓	✓	✓	✓
**Size & Performance**						
Max Matrix Size	No limit (fails)	5,000 rows	No limit (client RAM)	2,400 rows	Unknown (server error)	Up to 10M cells
Clustering (5K × 2K)	**X** Running for (12+ min)	7+ min	~30 sec	**X** File size limit	**X** 502 Server Error	3 min
Clustering (10.9K × 108)	**X** Error (3.5 min)	**X** Row limit	~25 sec	**X** Row limit	**X** 502 Server Error	30 sec
**Input & Data**						
Missing Value Imputation	**X**	Basic (5)	**X**	**X**	**X**	Advanced (8) + auto
**Visualization**						
Interactive (zoom/pan/hover)	✓	✓	**X** Static	**X** Static	✓	✓
						
**Interactive Features**						
Metadata Filtering	✓ (GUI)	**X**	**X**	**X**	✓(GUI)	✓(NLP)
Natural Language Interface	**X**	**X**	**X**	**X**	**X**	✓
GPU Acceleration	**X**	**X**	**X**	**X**	**X**	✓
**Downstream Analysis**						
Correlation Network	**X**	**X**	**X**	**X**	**X**	✓ (2D/3D)
Pathway Enrichment	**X**	**X**	**X**	**X**	✓(via Enrichr)	✓
PCA Analysis	**X**	**X**	**X**	✓	**X**	**X**
**Architecture**						
Parallelized Clustering	**X**	**X**	**X**	**X**	**X**	✓
**Accessibility**						
No Login Required	✓	✓	✓	✓	✓	✓
Actively Maintained	✓	✓	✓	✓	✓	✓

## Data Availability

The ClusterChirp source code is available on GitHub, with the front end at https://github.com/GumusLab/HeatmapFrontend and the heatmap backend at https://github.com/GumusLab/HeatmapBackend. Both repositories are released under the GNU Affero General Public License version 3.0 (GNU AGPL-3.0, https://www.gnu.org/licenses/agpl-3.0.en.html) and are also available under a commercial license for enterprises seeking additional features or wishing to avoid AGPL obligations. The tool is freely available at clusterchirp.mssm.edu.
