# Supplementary material for: ClusterChirp: A GPU-accelerated Web Server for Natural Language–Guided Interactive Visualization and Analysis of Large Omics Data
Source: ArXiv. 2026 Feb 9:arXiv:2602.08280v1. Preprint. [Version 1] (PMC12919222)
Supplement: Supplement 1 [file NIHPP2602.08280v1-supplement-1.pdf]

Supplementary Table

Table S1. Natural language commands that were tested.
Total Commands (n=45)

#	Command Type	Command	Expected Action
**1**	Filtering	show only male samples	Filter Gender = M
**2**	Filtering	filter to female samples	Filter Gender = F
**3**	Filtering	select responders only	Filter Response = Yes
**4**	Filtering	show non-responders	Filter Response = No
**5**	Filtering	filter to NE response	Filter Response = NE
**6**	Filtering	select samples at C1D1 timepoint	Filter Timepoint = C1D1
**7**	Filtering	show only C3D1 samples	Filter Timepoint = C3D1
**8**	Filtering	filter to C8D1	Filter Timepoint = C8D1
**9**	Filtering	select C12D1 timepoint	Filter Timepoint = C12D1
**10**	Filtering	show white patients only	Filter Race = white
**11**	Filtering	filter to asian samples	Filter Race = asian
**12**	Filtering	select black patients	Filter Race = black
**13**	Filtering	show hispanic samples	Filter Ethnicity = hispanic
**14**	Filtering	filter to non-hispanic	Filter Ethnicity = non_hispanic
**15**	Filtering	exclude male samples	Inverse filter Gender ≠ M
**16**	Filtering	remove C1D1 timepoint	Inverse filter Timepoint ≠ C1D1
**17**	Filtering	show male responders	Combined: Gender=M AND Response=Yes
**18**	Filtering	filter to female samples at C3D1	Combined: Gender=F AND Timepoint=C3D1
**19**	Filtering	show top 50 variant proteins	Variance-based filter
**20**	Filtering	clear all filters	Reset all filters
**21**	Clustering	cluster rows	Default row clustering
**22**	Clustering	cluster columns	Default column clustering
**23**	Clustering	cluster using Pearson correlation	Distance metric = Pearson
**24**	Clustering	cluster rows with Euclidean distance	Distance metric = Euclidean
**25**	Clustering	cluster using Manhattan distance	Distance metric = Manhattan
**26**	Clustering	cluster with cosine similarity	Distance metric = Cosine
**27**	Clustering	cluster using average linkage	Linkage = Average
**28**	Clustering	cluster with complete linkage	Linkage = Complete
**29**	Clustering	cluster rows using single linkage	Linkage = Single
**30**	Clustering	cluster genes using Pearson correlation and average linkage	Combined: Pearson + Average
**31**	Clustering	cluster samples by Euclidean distance and complete linkage	Combined: Euclidean + Complete
**32**	Clustering	cluster columns by Gender	Metadata-based clustering
**33**	Clustering	cluster samples by Timepoint	Metadata-based clustering
**34**	Clustering	cluster columns by Response	Metadata-based clustering
**35**	Sorting	sort rows alphabetically	Alphabetical sort (rows)
**36**	Sorting	sort columns alphabetically	Alphabetical sort (columns)

**37**	Sorting	sort proteins by variance	Variance-based sort
**38**	Sorting	sort samples by sum	Sum-based sort
**39**	Sorting	sort rows by variance descending	Descending variance sort
**40**	Sorting	sort samples by FASLG expression	Sort by specific protein
**41**	Sorting	sort columns by IL8 expression	Sort by specific protein
**42**	Sorting	sort samples by Gender	Metadata-based sort
**43**	Sorting	sort columns by Timepoint	Metadata-based sort
**44**	Sorting	sort samples by Response	Metadata-based sort
**45**	Sorting	sort columns by Race	Metadata-based sort

Table S2. Performance metrics for the natural language AI interface.

Metric Category	Metric	Result
**Command Success**	Overall Success Rate	95.66%
	Filtering Commands	96%
	Clustering Commands	98%
	Sorting Commands	93%
**Response Time Performance**	Average Command Processing Time	2 sec
	Clustering Operation Time	30 sec (For 10,000 rows)
**Reliability**	Commands Requiring Rephrasing	4%
	API Fallback Rate	6%
